# Maturation and Emigration of Single-Positive Thymocytes

**DOI:** 10.1155/2013/282870

**Published:** 2013-09-29

**Authors:** Xi Xu, Shusong Zhang, Pingping Li, Jin Lu, Qun Xuan, Qing Ge

**Affiliations:** ^1^Key Laboratory of Medical Immunology, Department of Immunology, Peking University Health Science Center, Ministry of Health, 38 Xue Yuan Road, Beijing 100191, China; ^2^Peking University Institute of Hematology, People's Hospital, Beijing, China; ^3^Department of Microbiology and Immunology, Kunming Medical University, Kunming, China

## Abstract

T lymphopoiesis in the thymus was thought to be completed once it reaches the single positive (SP) stage, a stage when T cells are “fully mature” and waiting to be exported at random or follow a “first-in-first-out” manner. Recent evidence, however, has revealed that the newly generated SP thymocytes undergo a multistage maturation program in the thymic medulla. Such maturation is followed by a tightly regulated emigration process and a further postthymic maturation of recent thymic emigrants (RTEs). This review summarizes recent progress in the late stage T cell development. The regulation of this developmental process is discussed.

## 1. Introduction

The thymus provides a unique microenvironment for the development and maturation of T cells. On the basis of CD4 and CD8 expression, T lymphopoiesis can be roughly divided into three major stages, namely, double-negative (DN), double-positive (DP), and single-positive (SP) cells. The key events during the process include the entry of lymphoid progenitor cells from bone marrow into the thymus and their differentiation to T cell precursors, the formation of functional T cell receptor (TCR) through TCR *β*-chain and *α*-chain rearrangement, and positive and negative selections to ensure the major histocompatibility complex (MHC) restriction to self-peptide as well as the clearance of autoreactive cells [1–4]. After highly regulated developmental process in the thymus, only about 1% of the thymocytes are able to emigrate and join the peripheral lymphocyte pool [5, 6].

Recently, many efforts have been made to reveal the dynamic and eventful development of SP cells in the thymic medulla. After positive selection, the newly generated CD4 or CD8 SP T cells migrate from the thymic cortex to the medulla, where they undergo negative selection as well as phenotypic and functional maturation [7–10]. During negative selection, strong interactions between TCR and self-peptide-MHC favor T cell apoptosis. T cells expressing TCRs with moderately high affinity to self-peptide/MHC are permissive for the development of naturally occurring effector T cells, such as natural regulatory T (nTreg) cells, natural killer T (NKT) cells, natural IL-17-producing T (nTh17) cells, and CD8*αα* ^+^ natural intraepithelial (nIEL) cells [11, 12]. T cells with low affinity TCRs survive the selection, tune down their responses to the same self-peptides, acquire functional competence, migrate out of the thymus, and enter the peripheral T cell pool. The thymic emigrants continue their post-thymic education in the periphery until at an unknown point, these cells become fully licensed to be mature resident naïve T cells. Thymic epithelial cells and dendritic cells are the main populations that promote the maturation of T cells after positive selection. This review will mainly discuss the maturation and migration of positively selected T cells in and outside of the thymus. Notably, most of the data were obtained in CD4 SPs. We will thus focus our effort on summarizing the research on CD4^+^ T cells. Whether the maturation and egress of CD8 SP follow a similar path remains to be seen. Naturally occurring effector T cells will not be discussed in the current review as nice summaries can be found elsewhere [13, 14].

## 2. Phenotypic and Functional Maturation of SP Thymocytes

CD4 or CD8 SP thymocytes are heterogeneous based on the expression of many molecules on the cell surface [15–17]. According to the expression of heat-stable antigen (HSA, or CD24), SP cells can be divided into two subgroups. The subgroup with CD24^lo^ was found to be functionally more mature than the one with CD24^hi^ [18]. 6C10 is expressed on DP and some SP cells [19]. Similarly, only positively selected DPs and a part of TCR^+^ SP cells express CD69 [20]. The acquisition of Qa2 and CD62L was used to identify a subset of mature SP thymocytes [8, 21]. Thus, based on the differential expression of these markers, multiple developmental pathways of SPs can be suggested. This will facilitate the studies of functional maturation of thymocytes after positive selection, the divergence of conventional T, nTreg, and nTh17 development, and the stages at which negative selection occurs.

For instance, a two-stage scheme was proposed with CD69^+^HSA^+^Qa2^−^ as the early stage of SPs that barely responded to Con A or anti-CD3 stimulation and CD69^−^HSA^−^Qa2^+^ as the late stage ones that responded to these stimuli by proliferation and cytokine secretion [15, 16, 19, 22]. However, studies finding an intermediate functional stage (CD69^−^Qa2^−^) or studies using other surface markers suggested that this two-stage scheme may be oversimplified [23, 24].

Based on the expression of chemokine receptor CCR7, two kinds of three-stage developmental models were used to investigate negative selection and the development of conventional T cells and nTregs [25, 26]. The first one combined the differential expression of CD24 and CCR7 and suggested that CD4 SP cells could be divided into three subsets: SP1 (CD24^+^CCR7^−^), SP2 (CD24^+^CCR7^+^), and SP3 (CD24^−^CCR7^+^). The second one combined CD69, CCR9 with CCR7 and suggested a slightly different three-stage SP development: CD69^+^CCR7^−/lo^CCR9^+^, CD69^+^CCR7^+^CCR9^−^, and CD69^−^CCR7^+^CCR9^−^. These developmental programs were confirmed by a gradual reduction of GFP intensities in these subsets in RAG2p-GFP transgenic mice. In these mice, the GFP expression is controlled by RAG2 promoter and the lingering GFP can be used as a molecular timer after RAG expression is terminated. Further studies showed that both models were in accordance with the down-regulation of CD24 and upregulation of Qa2 and CD62L [25, 26]. However, no functional comparisons were performed among these subsets. 

**Figure 1:**
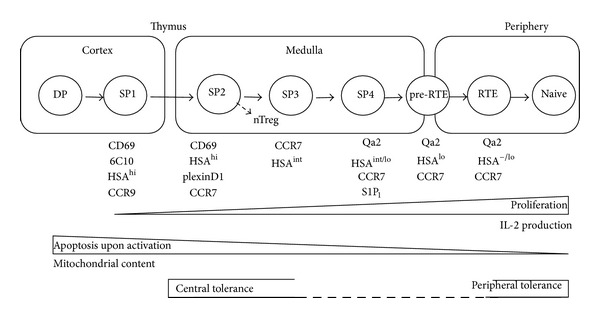
A schematic illustration of the development and migration of CD4 single-positive thymocytes and CD4^+^ recent thymic emigrants. DP: double-positive; SP, single positive; RTE: recent thymic emigrants; pre-RTE: thymus-leaving RTE precursors.

Based on the differences of CD69, 6C10, and Qa2 expressions, we resolved TCR*αβ* ^+^CD4^+^CD8^−^ thymocytes into four subsets: SP1 (6C10^+^CD69^+^), SP2 (6C10^−^CD69^+^), SP3 (CD69^−^Qa2^−^), and SP4 (CD69^−^Qa2^+^) (Figure 1) [9]. The chronological appearance of these subsets during mouse ontogeny and after the intrathymic adoptive transfer of SP1 cells has confirmed that these four subsets define a sequential and irreversible multistage program [9, 10]. From the progression of SP1 to SP4 thymocytes, a steady increase of proliferation and cytokine production upon Con A or anti-CD3/anti-CD28 stimulation was revealed (Figure 1) [9, 10]. An upregulation of the expression of costimulatory molecules, cytokine receptors, and transcription factors that regulate immune responses was also observed from the transcriptome comparison of these four SP subsets via microarray analysis [27]. Notably, cells with the most mature functions were found in SP4 subset, in accordance with the phenotypic maturation process and the appearance during ontogeny. Thus, a developmental blockage at the SP3-to-SP4 transition (shown in mice deficient in autoimmune regulator (Aire) or v-rel reticuloendotheliosis viral oncogene homolog B (Relb)) may result in defects in the functional maturation of CD4 SPs [9]. Indeed, compared to the thymic emigrants in wild type mice, the IL-2 secretion was significantly lower in Aire^−/−^ emigrants (Personal communication with Rong Jin).

In addition to the acquisition of immunocompetence, the functional maturation of SP thymocytes also includes an active “developmental tuning” process in which the signaling pathways of SPs are attenuated to respond appropriately to the selecting self-peptides in the periphery. Multiple intrinsic molecular changes, such as altered subcellular distribution and phosphorylation sites of Lck [28, 29], altered expression of inhibitory coreceptor CD5 [30], altered glycosylation of cell-surface receptors [31], and altered microRNA profiles [32], may all contribute to the tuning of TCR threshold. However, at what developmental stage(s) this maturational tuning occurs and what mechanisms regulate such tuning remain elusive.

## 3. Migration of SP Thymocytes

The identification of these intermediate stages makes it possible to dissect the cellular and molecular mechanisms underlying the differentiation of CD4 SP thymocytes and facilitates the study of how microenvironments of various thymic niches modulate the SP maturation. For instance, by comparing the expression of molecules associated with cell migration, we found that the cortex-to-medulla migration may not occur immediately after positive selection and the generation of SPs [27]. SP2, but not the newly generated SP1 thymocytes, had a downregulated CCR9 and upregulated plexinD1 and CCR7 expressions (Figure 1). CCL25, the ligand of CCR9, is produced by cortical thymic epithelial cells, thus retaining CCR9^+^ DP and SP1 thymocytes in the cortex [33]. This CCR9/CCL25 signaling can be suppressed by plexinD1 [34]. The medullary migration of SPs is regulated by CCR7 [7, 35–37]. The deficiency of *Plxnd1*, *Sema3e* (a functional ligand for plexinD1), or *Ccr7* leads to the accumulation of SP thymocytes in the cortex. Thus, the upregulation of plexinD1 and CCR7 in SP2 thymocytes promotes these cells migrating toward the medulla [25, 27]. As the appearance of nTreg precursors was detected in CD69^+^CCR7^+^CCR9^−^ CD4 SPs, it also suggests that the divergence of nTreg and conventional T cells may start at the SP2 stage in the thymic medulla [25]. Interestingly, the mRNA levels of Forkhead box O1 (FoxO1) and Kruppel Like Factor 2 (KLF2), known as transcription factors that regulate the expression of egress-enabling molecule sphingosine 1-phosphate receptor type 1 (S1P_1_) [38–41], were also significantly upregulated in SP2 cells, or CD69^+^CCR7^+^CCR9^−^ CD4 SPs [25, 27]. It is not clear why these changes do not occur in the newly generated CD69^+^ SP1 cells as the changes of these molecules were thought to come from positive selection-derived signals [35]. Signals from the medullary microenvironment may be also involved in regulating such delayed expression.

The requirement of CCR7 for the accumulation of CD4 SP cells in the medulla was confirmed by direct visualization of the migration pattern of purified SPs within thymic slices or cut thymic preparations using a two-photon microscopy [42, 43]. This technology offers a powerful tool to study the migration and cell-cell interaction of SPs in the thymic medulla. Using this approach, the movement of medullary thymocytes was found to be rapid and follow confined migratory paths. Such migration was slowed down and restricted in the presence of a negative selecting ligand. The application of CD11c-YFP reporter mice further revealed that thymocytes and dendritic cells made frequent and transient interactions, supporting the widely accepted view that SPs scan self-antigens on epithelial cells and dendritic cells in the thymic medulla. 

Interestingly, the residence time of SP cells in the thymic medulla is in the order of days. For instance, the residency time of 4–7 days was suggested by BrdU pulsing study, intrathymic adoptive transfer of SP1 cells, or intrathymic delivery of recombinant adenoviruses expressing MHC class II molecule into MHC II deficient mice [10, 15, 44]. Based on the decay of GFP intensity on SP thymocytes in RAG2p-GFP transgenic mice, a shorter and narrower window of 4-5 days of medullary persistence time was calculated [8]. Despite the differences in suggested residency time, it remains to be determined whether these many days are entirely required for the removal of strong responsiveness to self-ligands. The acquisition of functional competence and egress capability may also take days to accomplish. 

## 4. Negative Selection

SP thymocytes in the thymic medulla must survive negative selection before exit. It is a process in which thymocytes bearing strong self-reactivity TCRs undergo apoptosis. Negative selection prevents the maturation and emigration of strongly self-reactive T cells and subsequent potential to mount an autoimmune response in the periphery. Thus, the increase of autoreactive T cells in the periphery with the potential to cause autoimmunity was often observed in mice lacking the negatively selecting self-peptide-MHC complexes in the medulla [45, 46], or with defects in T cell migration toward the thymic medulla, or in mice with disorganized thymic medulla. Examples include mice deficient in CCR7 [7], Relb [47–49], NF-*κ*B2 [50], NIK [51], lymphotoxin *β* receptor (LT*β*R) [52], TRAF6 [53], CD40 [54, 55], or Aire [9, 56–58]. 

The medullary thymic epithelial cells (mTECs) play a particularly important role in negative selection as the promiscuous expression of tissue-specific antigens (TSAs) within the thymus was found to be enriched in mTECs, in contrast to cortical thymic epithelial cells (cTECs), dendritic cells, and macrophages [59]. To date, the only identified transcription factor that regulates the promiscuous gene expression of TSA in mTECs is Aire. Its critical contributions to central tolerance were realized since the identification of the *AIRE* gene in 1997 [60, 61]. Defects in Aire expression result in autoimmune-polyendocrinopathy-candidiasis-ectodermal dystrophy (APECED) in human and multiorgan autoimmunity and serum autoantibodies in mice [57, 62–64]. The regulation of TSA expression in mTECs is mediated by the interaction of Aire oligomers and positive transcription elongation factor b (P-TEFb), bringing the latter to RNA polymerase II (RNAPII). The phosphorylated RNAPII is then competent for elongation and cotranscriptional processing of target genes, leading to the expression of TSAs and their presentation to T cells via MHC [65, 66]. In addition to the regulation of TSA expression and tolerance induction, the antigen processing in mTECs can also be affected by Aire [67]. Notably, Aire is also involved in the regulation of thymocyte and dendritic cell migration. The expressions of CCR4, CCR7, and XCR1 ligands in the medullary epithelial cells were modulated by Aire [68, 69]. Thus, the deficiency of Aire resulted in diminished emigration of mature CCR7-expressing SP thymocytes and diminished accumulation of dendritic cells in the thymus.

Appropriate differentiation of mTECs is essential for Aire-dependent and -independent TSA expression and negative selection. Lacking a signaling component Sin, or perturbations of NF-*κ*B signaling, such as in mice deficient in LT*β*R, its ligands LT*β* or LIGHT, CD40, and RANK, all result in defects in mTECs maturation, Aire expression, and defective negative selection [52, 54–56, 70].

The mechanisms of endogenous self-antigen loading onto MHC class II in mTECs were not clear until recently. The shuttling of intracellular antigens onto MHC class II by macroautophagy was first obtained in several cell culture models [71–75]. Its physiological relevance was then examined in the thymus. Macroautophagy-positive mTECs bear a mature CD80^hi^MHCII^hi^ phenotype and highly express Aire and TSAs [59, 76]. The direct evidence of macroautophagy in MHC II loading of endogenous antigen for negative selection was provided by Aichinger et al. [77]. They used PCC and GFP-CRP-LC3 transgenic mouse lines to directly prove *in vivo* the essential role of macroautophagy in self-antigen loading in mTECs. Using mice reconstituted with MHC II-deficient BM and with thymi transplantation, they further provided evidence that Aire-driven TSAs might be presented directly by mTECs themselves [77]. Transplantation of thymi lacking autophagy gene *Atg5* resulted in the escape of forbidden CD4 T cell-specificities.

In addition to mTECs' direct presentation of TSAs to SPs in the medulla, medullary DCs (mDCs) can acquire TSAs via the uptake of apoptotic mTECs and indirectly present them on the cell surface [78–80]. Selective depletion of thymic DCs in a transgenic mouse model resulted in an increased frequency of CD4 SP T cells in the thymus and consequential autoimmunity [81]. mDCs in the thymus can be divided into two subsets, namely, conventional DCs (cDCs) and plasmacytoid DCs (pDCs). Based on the expression of CD8*α* and signal-regulatory protein (sirp)*α*, cDCs can be further divided into CD8*α* ^+^sirp*α* ^−^ cDCs and CD8*α* ^−^sirp*α* ^+^ cDCs [82, 83]. While CD8*α* ^+^sirp*α* ^−^ cDCs arise from intrathymic precursors, both CD8*α* ^−^sirp*α* ^+^ cDCs and pDCs migrate from the peripheral blood. These thymus-homing peripheral DCs promote central tolerance by bringing blood-borne antigens to SPs [84–87].

 Although accumulating evidence has established that central tolerance occurs in the thymic medulla, its impact on the maturation process of SP thymocytes remains unclear. In mice with defects in negative selection, such as RelB^−/−^ and Aire^−/−^ mice, a developmental blockage was observed between SP3 and SP4 thymocytes. This may implicate that the SP3/SP4 transition could be a critical checkpoint for SP cell development and negative selection [9]. Similarly, McCaughtry et al. also found that only HSA^hi^Qa2^−^ SP thymocytes were competent to undergo clonal deletion [8]. Recently, Daley et al. revealed that the Ikaros family transcription factor Helios was specifically induced in Foxp3^−^ CD4 SP cells undergoing negative but not positive selection. The expression of Helios together with the proapoptotic protein Bim marked the first wave of thymic deletion in CCR7^−^CD4^+^CD69^+^ thymocytes. The second wave of removing autoreactive T cells occurred in CCR7^+^CD4^+^CD69^+^ thymocytes with the expression of Helios and activation of Card11 and c-Rel (Figure 1) [26]. This kind of “activation” made the cells resistant to Bim-mediated apoptosis in the absence of actual activation and cell proliferation as no expression of growth mediators such as IL-2 and Myc could be detected in Helios positive SP thymocytes. It is not clear whether negative selection of strongly self-reactive CD4 SP can be accomplished before the down-regulation of CD69 and upregulation of Qa2 in SPs. If not finished, whether helios is also involved in the central tolerance of relatively mature SPs and how SPs are signaled to stop scanning of self-peptides presented by mTECs and mDCs remain to be investigated.

## 5. Redox Regulation of SPs

The transition from developing T cells in the thymus to mature T cells in the periphery marks a dramatic change in their environment. Factors in the blood, secondary lymphoid organs, or even nonlymphoid tissues may have an impact on T cells during their emigration. For instance, the oxygen tension in blood was found to be 5–13 kPa [88], dramatically higher than the oxygen tension in the thymus (1.3 kPa) [89]. A modulation of redox balance and/or reduction of mitochondrial content may be required to prepare mature thymocytes for the increased oxygen level in the periphery. Indeed, a decrease of mitochondrial content was found in CD8 SP thymocytes when compared to DP cells [90]. The amount of mitochondria was further reduced in the peripheral CD8^+^ naïve T cells. High mitochondrial content leads to the production of excessive ROS, a proapoptotic factor for T cells [91, 92]. High mitochondrial level also induces the intrinsic death pathway mediated by Bcl-2 family proteins [93]. Thus, in Atg7^−/−^ mice, an increase of mitochondrial content and enhanced cell apoptosis was found in CD8^+^ T cells. The data further suggested that Atg7-mediated autophagy plays an essential role in the clearance of mitochondria during late stage thymocyte maturation.

Interestingly, similar levels of mitochondria were found between CD4 SP thymocytes and DP cells. Yet reduced mitochondria level was detected in naïve CD4^+^ T cells in the lymph nodes [90]. This indicates that the removal of mitochondria in CD4^+^ T cells may occur later than CD8^+^ ones, that is, during late stage SP development and/or after thymic egress. In addition, we found a significant increase of ROS in the most mature CD4^+^ SPs, Qa2^+^CD69^−^ SP4 cells. The level of ROS in this SP subset was very similar to that in mature naïve CD4^+^ T cells (Jin et al., Immunology and Cell Biology, in press). It was reported that in *Ncf*1^−/−^ mice, the reduced ROS level may allow the survival and egress of autoreactive thymocytes, thus causing autoimmune diseases [94]. This implies that the prooxidant shift in late stage SP cells may facilitate negative selection. ROS was also shown to induce autophagy via autophagy gene Atg4 [95]. It would thus be interesting to investigate whether this increase of ROS in Qa2^+^ going-to-be-thymic emigrants initiates the autophagy to remove excessive mitochondria and promote the survival of thymic emigrants outside the thymus.

## 6. Emigration of Mature SP Thymocytes

After completing the developmental program, mature SP thymocytes leave the thymus as recent thymic emigrants (RTEs) and join the population of peripheral lymphocytes. The export ratio of RTEs to total thymocytes is kept constant during lifetime in healthy individuals, although the absolute number changes when thymus involutes during aging [5, 96–98]. Constant thymic exportation helps maintain the diversity of peripheral TCR repertoire and promote the recovery from lymphoablation [99–101].

Multiple approaches have suggested that SPs with a phenotype of CD69^−^HSA^lo^Qa^2+^ (SP4) acquire the thymic egress capability. Using intrathymic injection of fluorescein isothiocyanate (FITC) and Rag2p-GFP transgenic mice, very similar phenotypes were found between SP4 thymocytes and RTEs leaving the thymus within a week [8, 102].

At the molecular level, SP4 cells were found to express the highest levels of sphingosine-1-phosphate (S1P) receptor, S1P_1_ and CD62L compared to other immature SP subsets, enabling them to leave the thymus and migrate towards lymph nodes [27]. The S1P and S1P_1_ play a dominant role in regulating thymocyte emigration. S1P_1_ is expressed by mature SP cells, while S1P is produced by vascular endothelium as well as neural crest-derived pericytes that ensheathe the blood vessels [6, 103–105]. The high concentration of S1P in blood and around perivascular space (PVS) attracts S1P_1_-expressing mature thymocytes to export [6, 106]. Mice deficient in S1P_1_, sphingosine kinases (essential for the production of S1P), or lipid phosphate phosphatase 3 (LPP3 can degrade thymic S1P) showed a significant decrease of thymic output and accumulation of thymocytes in the thymic medulla [6, 104, 105, 107]. The agonist of S1P, FTY720, inhibits the egress of thymocytes from the thymus [108–110]. Other factors, such as KLF2 (transcription factor of S1P_1_), PI3 K (negative regulator of KLF2) and PTEN (negative regulator of PI3 K) can also influence thymocyte emigration [40, 111, 112]. Other molecules, such as CCR7, CXCR4, early growth response gene 1 (Egr1) [113], aryl hydrocarbon receptor (AHR) [114], laminin-5 [115], and integrin *α*5*β*1 [116], have also been demonstrated to be involved in the process. However, it remains elusive whether positive selection alone or together with signals from thymic medulla drives SPs to leave the thymus and whether negative selection and maturation have an impact on the egress.

The export of thymocytes to the periphery is believed to occur mainly through PVS. Various groups have reported the presence of lymphocytes in the PVS by transmission electron-microscopic (TEM) and scanning electron-microscopic (SEM) studies. Giant PVS filled with thymocytes was also found in the nonobese diabetic (NOD) mice [117–120]. More direct evidence was revealed by intravascular injection of PE-conjugated anti-CD4 antibody. Within 5 minutes of the antibody injection, CD4^+^ T cells in the PVS can be selectively labeled. The results indicated that the majority of thymocytes emigrate via PVS at the cortico-medullary junction [6]. In or around the PVS region, the thymus-leaving RTE precursors (pre-RTEs) may continue interacting with DCs and upregulating the expression of their maturation marker, Qa2 (Figure 1) [102]. Thus, in Aire^−/−^ mice in which thymic epithelial cells failed to support the Qa2 upregulation in the thymus, the interaction of SPs with DCs around the PVS may facilitate the expression of Qa2 in emigrating cells. Whether negative selection occurs at this stage is currently not known. At least one study found that in CCR2^−/−^ mice, the thymic Sirp*α* ^+^ cDC subset was decreased in the PVS, and a modest impairment in intrathymic negative selection against blood-borne antigens was revealed [87].

## 7. Post-Thymic Maturation of RTEs

RTEs were regarded having the same properties as mature naïve T cells until the 1970s, when it was first proposed that T cells leave the thymus in an immature state and complete their development in the periphery [121]. Subsequent studies further revealed that RTEs and peripheral mature naïve T cells are different not only in phenotype but also in function [9, 97, 122]. For instance, using RAG2p-GFP transgenic mice or FITC/BrdU labeling, a downregulation of CD24 and CD3/TCR and upregulation of Qa2, CD28, CD45RB, IL-7R*α*, and Ly6C were found in CD4^+^ RTEs as they gradually mature into naïve T cells [22, 122–126]. Compared to naïve CD8^+^ T cells, CD8^+^ RTEs express higher level of *α*4*β*7, *α*E integrin, and CCR9 [127–129].

In accordance with phenotypic differences, RTEs and naïve T cells are functionally distinct. Upon activation, CD4^+^ RTEs revealed diminished proliferation, less production of IL-2, IL-4, and interferon-*γ* (IFN-*γ*), and lower expression of CD25 when compared to naïve CD4^+^ T cells [122, 130, 131]. Under Th1, Th17, and iTreg polarizing conditions, CD4^+^ RTEs expressed less characteristic cytokines or major transcription factors. Compared to CD8^+^ naïve T cells, activated CD8^+^ RTEs produced less cytokines and generated fewer IL-7R*α* ^hi^KLRG1^lo^ memory precursor effector cells [122, 132–134].

Another important difference lies in the TCR repertoire between RTEs and naïve T cells. Complementarity determining region 3 (CDR3) length spectratyping revealed that TCRs expressed by RTEs were skewed toward longer CDR3 regions, suggesting the existence of more autoreactive cells in the population of RTEs [135–137]. Thus, peripheral tolerance of RTEs may be a necessary extension of negative selection in the thymus [138, 139]. Self-antigens presented by DCs and lymphoid stroma in the periphery are believed to play an important role in promoting peripheral tolerance [140, 141]. Although lacking direct evidence, the higher expression of *α*4*β*7, *α*E integrin, and CCR9 may facilitate CD8^+^ RTEs homing to the gut-associated lymphoid tissues and gaining tolerance to self-antigens and harmless food antigens [127–129]. The diminished proliferation, defective cytokine secretion, and the expression of inhibitory receptors such as CTLA-4 and PD-1 may also help prevent autoreactive RTEs from harmful tissue damage [97, 122, 123, 142].

The above evidence all suggests that RTEs are a unique T cell population. However, the key events in the transition of RTEs to mature naïve T cells and its regulation are largely unknown. Peripheral lymphoid tissue, in particular dendritic cell compartment, is indispensable for the maturation of RTEs whereas self peptide-MHC complexes and IL-7 are less important [143, 144]. The transcriptional repressor NKAP may also influence RTE maturation, as its deficiency keeps RTEs from full maturation [145].

In addition, RTEs are widely distributed in the periphery, such as lymph nodes, Peyer's patches, spleen, blood, and small intestine of mice [122, 129, 146]. Notably, it was also found that RTEs migrate into the autoimmune thyroid disease glands or the colonic mucosa in ulcerative colitis patients [147, 148]. Thus, the microenvironments of different tissues at physiological or pathological conditions may also affect the maturation of RTEs.

## 8. Concluding Remarks

Accumulating evidence has suggested that SP thymocytes and RTEs are not simply waiting in line to become mature naïve T cells. The maturation and migration of these T cell populations are essential for the establishment and maintenance of a self-tolerant, diverse, and functional T cell repertoire. The eventful maturation of SPs in the thymus and that of RTEs in the periphery and the tolerance induction of these cells may be connected by the same migratory dendritic cell populations. However, microenvironments in the thymus and in various lymphoid tissues in the periphery may shape the T cell pool in a different way and give unique imprints in the acquisition of T cell functions as well as migratory properties. Peripheral infection and inflammation may also change the maturation process of RTEs. Compared to our knowledge of the early stages of thymocyte development, the detailed process and regulation of SP-RTE-Naïve T cell transition are far from being understood. The underlying mechanisms of this program and their significance in diseases should be further explored.
